# Rational decisions or moral obligations? A systematic review of TPB-NAM integration in understanding low-carbon behavior

**DOI:** 10.3389/fpsyg.2026.1894328

**Published:** 2026-08-18

**Authors:** Bing Wei, Yudi Fernando, Peng Tian, Hui-E Lu, Thurasamy Ramayah, Muhammad Shabir Shaharudin

**Affiliations:** 1 School of Management, Universiti Sains Malaysia, Gelugor, Malaysia; 2 College of Food and Quality Engineering, Nanning University, Nanning, China; 3 Faculty of Industrial Management, Universiti Malaysia Pahang Al-Sultan Abdullah, Gambang, Malaysia

**Keywords:** environmental psychology, intention-behavior gap, low-carbon behavior, norm activation model, theory of planned behavior

## Abstract

**Objective:**

Understanding low-carbon behavior remains a significant challenge due to the complexity of environmental decision-making and the persistent intention–behavior gap. While the Theory of Planned Behavior (TPB) and the Norm Activation Model (NAM) are among the most widely applied frameworks for explaining environmental behavior, studies increasingly integrate these theories to capture both rational and moral dimensions of decision-making. This study systematically reviews TPB–NAM integration research to identify major research trends, theoretical integration approaches, and implications for understanding the psychological mechanisms underlying low-carbon behavior.

**Methods:**

A systematic review was conducted using 225 peer-reviewed articles retrieved from the Web of Science Core Collection. Bibliometric and content analysis techniques were employed using Bibliometrix, VOSviewer, and Scimago Graphica to examine publication trends, research hotspots, application contexts, dependent variables, and integration pathways between TPB and NAM.

**Results:**

The findings reveal a substantial increase in TPB–NAM integration studies since 2019, reflecting growing interest in environmentally responsible behavior. Sustainable consumption, waste management, and sustainable transportation emerged as the most frequently investigated contexts. Behavioral intention remained the dominant dependent variable, accounting for 85.4% of studies, whereas actual behavior received comparatively limited attention. Four major integration pathways were identified, namely awareness of consequences → attitude, subjective norm → personal norm, awareness of consequences → subjective norm, and social norm → personal norm. Furthermore, 62.0% of studies adopted a simple combination of TPB and NAM without establishing clear theoretical linkages between constructs.

**Conclusion:**

The findings suggest that TPB and NAM represent complementary rather than competing perspectives for understanding environmental behavior. The identified integration pathways highlight the interaction of cognitive evaluation, social influence, and moral regulation in shaping low-carbon behavior. By synthesizing existing evidence, this review advances understanding of the psychological foundations of environmental behavior and provides directions for future research aimed at addressing the intention–behavior gap and promoting sustainable behavioral change.

## 1 Introduction

**Figure 1:**
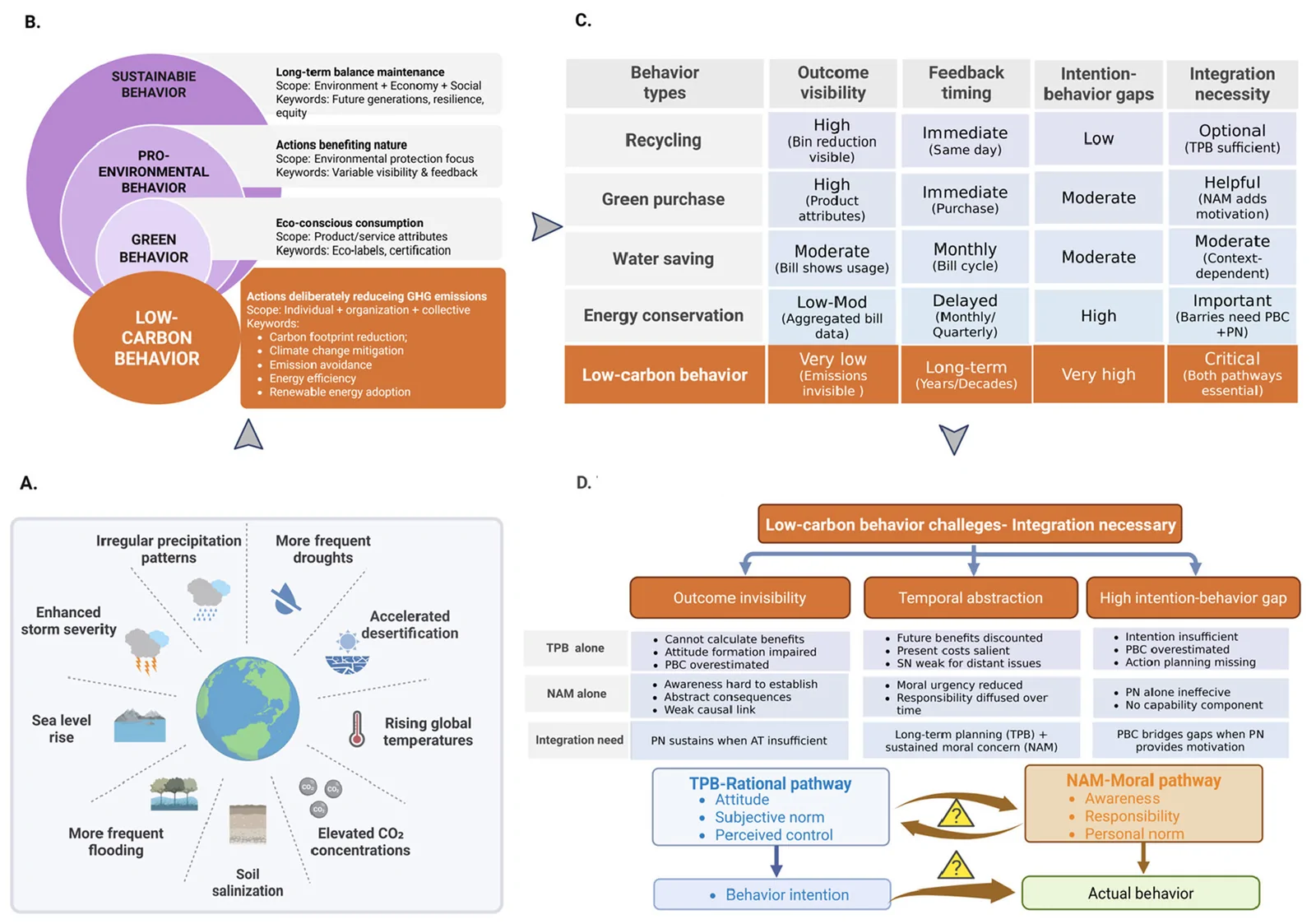
From climate change to theoretical integration: a narrative framework for understanding low-carbon behavior. **(A)** Key environmental impacts of climate change. **(B)** Environment behavior taxonomy. **(C)** Low-carbon behavior distinctiveness. **(D)** TPB-NAM integration in low-carbon context. Created in BioRender. Wei, B. (2026) https://BioRender.com/2bv3v6h.

Climate change has emerged as one of the most pressing environmental crises of the 21st century, with human activities contributing substantially to carbon emissions (Saxena, 2025; Tissaoui and Zaghdoudi, 2025). Addressing this challenge requires fundamental changes in human behavior across multiple domains, from energy consumption to transportation choices. As climate impacts intensify—manifesting through irregular precipitation, rising temperatures, and accelerated environmental degradation—understanding and promoting pro-environmental behavior has become a critical research agenda for achieving sustainability (Figure 1A). Many studies have attempted to improve environmental behavior from psychological and technological perspectives (Abu Elsamen et al., 2025; Neves et al., 2025; Yan et al., 2025), as documented in prior systematic reviews of this literature (e.g., Colombo et al., 2023; Topal et al., 2021). However, with the deepening development of environmental behavioral research, researchers increasingly recognize the inherent limitations of single theoretical frameworks in explaining human behavioral decision-making mechanisms (Cox and Santos, 2025; Hu et al., 2026; Soni and Bhukya, 2025; Tripathi and Trigunait, 2026).

Two theoretical frameworks have dominated environmental behavior research: the Theory of Planned Behavior (TPB) and the Norm Activation Model (NAM). TPB, developed by (Ajzen 1985), explains rational decision-making through attitudes, subjective norms, and perceived behavioral control. While effective for understanding deliberate choices, TPB often falls short in addressing the moral dimensions of environmental behavior (Al Mamun et al., 2024; Mirabella et al., 2025; Song et al., 2024; Wong et al., 2024). Meta-analytic research reveals that TPB demonstrates reduced explanatory power for pro-environmental behaviors (Bamberg and Möeser, 2007). This limitation becomes particularly pronounced when moral dimensions are not adequately captured. Conversely, NAM, proposed by (Schwartz 1975), explains prosocial behavior through personal norms activated by awareness of consequences and ascription of responsibility. Despite its strength in capturing moral motivation, NAM is limited by its focus on norm activation while neglecting rational decision processes and perceived behavioral control (Bamberg and Schmidt, 2003; Khan et al., 2025; Zhang Y. et al., 2024). Empirical studies show moral norm activation alone proves insufficient when practical implementation barriers exist (Klöckner, 2013). Research demonstrates that for behaviors requiring sustained effort and resources, moral obligation without accompanying efficacy beliefs leads to frustration rather than action (Klöckner, 2013). These complementary limitations suggest that neither theory alone can fully explain environmental behavior formation mechanisms.

Among various environmental behaviors, low-carbon behavior—deliberate actions taken by individuals, organizations, or social entities that reduce greenhouse gas emissions through changes in consumption patterns, production processes, or participation in environmental initiatives (Wei and Shaharudin, 2025; Whitmarsh et al., 2025)—has attracted growing scholarly attention as a critical pathway for climate change mitigation. This multi-level phenomenon encompasses individual daily choices (e.g., energy conservation, transportation), organizational operational decisions (e.g., production optimization, supply chain management), and social collective actions (e.g., policy support, public participation) (Wei and Shaharudin, 2025). Yet, this behavior poses unique challenges for theoretical explanation. Unlike recycling or green purchasing, which provide immediate and visible feedback, carbon reduction presents a striking contradiction: it demands sustained commitment while offering little tangible return. Emissions avoided cannot be seen, heard, smelled, or touched—violating assumptions of perceivable outcomes that underpin TPB's rational calculation framework (Figure 1B). Climate benefits accrue over years, not moments, creating temporal discounting effects that weaken both rational incentives (TPB) and moral urgency (NAM; Figure 1B). Most critically, low-carbon behavior exhibits substantial intention-behavior gaps—research consistently documents substantial gaps between climate awareness and behavioral adoption (de Matos et al., 2025; Kaiser and Brüggemann, 2025; Stern, 2000), with longitudinal studies revealing that while attitudes, subjective norms, and perceived behavioral control significantly predict intentions, the translation of these intentions into behavior remains problematic (Figure 1C). These characteristics systematically undermine single-theory explanations: TPB's rational calculation struggles when outcomes remain invisible, while NAM's moral activation weakens when consequences feel distant and abstract. It is precisely this explanatory difficulty that renders theoretical integration not merely useful, but theoretically necessary (Figure 1D).

The necessity of integrating TPB and NAM has been well-documented in the literature. Bamberg and Möeser's (2007) meta-analysis of 57 studies demonstrated that neither rational decision-making nor moral norm pathways alone can fully explain behavioral formation mechanisms. Their analysis revealed that while TPB explained 27% of pro-environmental behavioral variance, incorporating personal norms as a third predictor alongside attitudes and behavioral control substantially improved explanatory power, accounting for 52% of variance in behavioral intentions. (Steg and Vlek 2009) further emphasized that behavioral interventions based on single theoretical frameworks yield suboptimal effects. Building on this foundation, researchers have increasingly attempted to integrate TPB and NAM frameworks. (Klöckner 2013) demonstrated that integrated theoretical models combining both frameworks explained 36% of behavioral variance, 55% of intention variance, and 47% of personal norm variance across 56 diverse environmental behaviors, substantially outperforming single-theory approaches. (Kaiser 2006) enhanced explanatory power when integrating moral norms into TPB. Recent multi-disciplinary research has applied this integration across various domains including environmental protection (Borozan and Pfeifer, 2023; Ding et al., 2023), sustainable consumption (Phu et al., 2024; Severijns et al., 2023), and sustainable transportation (Abdoulaye et al., 2024; Mosca et al., 2024; Sheng and Zhang, 2022).

To systematically understand the current state of TPB and NAM integration research, we reviewed existing literature reviews in this area (Table 1). Analysis of these reviews reveals an evolution of research perspectives from conceptual model construction (Luelfs and Hahn, 2013) to broader domain applications including urban sustainability (Topal et al., 2021), tourism environmental behavior (Han, 2021), and the environmental knowledge-action gap (Colombo et al., 2023). Recent studies have further specialized into specific contexts such as e-waste recycling (Michael et al., 2024) and pandemic commuting (Zarabi et al., 2024). However, these reviews share three common limitations. First, they primarily employ qualitative analysis without systematic methods using quantitative social network approaches to reveal development trends. Second, they focus on specific contextual applications rather than examining theoretical integration patterns across domains. Third, and most critically, they lack analysis of how TPB and NAM constructs have been connected, leaving the field without clear guidance on integration approaches.

**Table 1:** Literature reviews of TPB and NAM integration research.

References	Methods/ software	Keywords
(Luelfs and Hahn 2013)	Literature review and conceptual model development	Voluntary pro-environmental behavior; corporate greening; psychological determinants; theory of planned behavior; norm-activation model; organizational citizenship behavior; literature review
(Topal et al. 2021)	Systematic literature review	urban sustainability understanding; sustainable behavior; pro-environmental behavior; awareness; perception; attitude; environmental concern; theory of planned behavior; value-belief-norm theory; new ecological paradigm
(Han 2021)	Literature review	Environmentally-sustainable consumer behavior; tourism and hospitality; key drivers of eco-friendly behavior; theories related to environmentally-sustainable behavior; environmental sustainability
(Colombo et al. 2023)	Literature review	Pro-environmental behavior; knowledge-action gap; self-regulation; executive functions
(Michael et al. 2024)	Quantitative approach with cross-sectional survey design using SmartPLS	E-waste; theory of planned behavior; recycling intention; willingness to pay; awareness
(Zarabi et al. 2024)	Systematic review of 36 scientific publications using the PRISMA 2020 method	Sustainable transport; travel behavior change; COVID-19; essential workers; pandemic commuting; mode choice

To address these gaps, this study conducts a systematic review of TPB and NAM integration research in environmental behavior. The objectives are: (1) to map the development trajectory and knowledge structure of this research field through publication trends, keyword co-occurrence networks, and geographic distribution analysis; (2) to systematically identify and categorize the patterns of theoretical integration between TPB and NAM constructs; (3) to map the specific pathways through which researchers have connected these two theories; and (4) to provide evidence-based guidance for future research, particularly for emerging areas such as low-carbon behavior that require sophisticated theoretical frameworks to address their unique characteristics of invisible outcomes, temporal abstraction, and substantial intention-behavior gaps as illustrated in Figure 1. These objectives correspond to three research questions: RQ1 (developmental trajectory), RQ2 (integration pathways), and RQ3 (implications for low-carbon behavior research).

The remainder of this paper is organized as follows. Section 2 presents the theoretical background of TPB and NAM, establishing the rationale for their integration. Section 3 describes the research methodology, including data collection, analytical tools, and content analysis procedures. Section 4 reports the bibliometric results, covering publication trends, keyword analysis, and geographic distribution. Section 5 discusses the integration patterns identified through content analysis. Section 6 concludes with theoretical and practical implications, limitations, and directions for future research.

## 2 Theoretical background

### 2.1 Theory of planned behavior

TPB posits that behavioral intention is the most proximal determinant of behavior, shaped by three factors: attitude toward the behavior (positive or negative evaluation), subjective norm (perceived social pressure), and perceived behavioral control (perceived ease or difficulty of performing the behavior) (Ajzen, 1985, 1991, 2011). Meta-analyses have demonstrated that TPB explains substantial variance in both intention and behavior (Armitage and Conner, 2001), with recent longitudinal research further confirming that perceived behavioral control moderates the intention-behavior relationship (Hagger and Hamilton, 2024).

However, TPB faces limitations in environmental behavior contexts. Meta-analytic research reveals that TPB demonstrates reduced explanatory power for pro-environmental behaviors, explaining approximately 27% of behavioral variance (Bamberg and Möeser, 2007). This limitation becomes particularly pronounced when moral dimensions are not adequately captured. Empirical studies of specific environmental actions demonstrate that integrated theoretical approaches combining rational and moral pathways achieve substantially higher variance explanation than TPB alone (Klöckner, 2013). Its emphasis on rational decision-making may underestimate moral and emotional factors driving pro-environmental actions (Harland et al., 1999; Urban et al., 2023). The subjective norm construct focuses on specific referent others, potentially missing broader moral norms. A critical theoretical limitation emerges when outcomes remain temporally distant and perceptually invisible: TPB's assumption of calculable cost-benefit tradeoffs becomes systematically challenged by environmental behaviors where benefits cannot be directly observed (Conner and Norman, 2022). Moreover, environmental behavior research consistently documents substantial intention-behavior gaps (Conner and Norman, 2022). These limitations suggest that TPB requires integration with complementary frameworks to fully capture environmental behavior formation.

### 2.2 Norm activation model

NAM focuses on moral influences on prosocial behavior. NAM suggests that behavior is driven by personal norms—internalized feelings of moral obligation—activated through two preconditions: awareness of consequences (AC, recognition that one's actions affect others or the environment) and ascription of responsibility (AR, acceptance of personal responsibility for these consequences) (Schwartz, 1975, 1977). When both conditions are met, personal norms are activated, generating moral obligation that motivates pro-environmental behavior (De Groot and Steg, 2009).

Recent research continues to validate NAM in environmental contexts. (Savari et al. 2023) demonstrated that the integrated NAM-TPB model explained 77% of variance in farmer pro-environmental behavioral intention, with awareness of consequences emerging as a key predictor. A systematic review of NAM applications (2018-2023) found that 98% of studies extended NAM by integrating additional constructs, confirming both its relevance and limitations (Jaffar and Latiff, 2024).

However, NAM's limitations include insufficient attention to practical constraints and self-efficacy beliefs (Klöckner, 2013), and its exclusive focus on personal norms may underestimate rational cost-benefit calculations (Bamberg and Schmidt, 2003). Empirical studies show moral norm activation alone proves insufficient when practical implementation barriers exist (Klöckner, 2013). Research demonstrates that for behaviors requiring sustained effort and resources, moral obligation without accompanying efficacy beliefs leads to frustration rather than action, revealing a fundamental theoretical gap: NAM's exclusive focus on moral imperatives (“ought”) overlooks the practical feasibility dimension (“can”), yet successful behavior requires both moral motivation AND perceived capability (Bamberg and Schmidt, 2003). These limitations suggest that NAM benefits from integration with frameworks addressing rational decision processes.

### 2.3 Rationale for integrating TPB and NAM

Given TPB's underestimation of moral dimensions and NAM's neglect of practical feasibility (as discussed above), empirical research has systematically tested whether integration overcomes these complementary limitations. Meta-analytic evidence confirms substantial gains: Bamberg and Möser (2007) found that incorporating personal norms into TPB nearly doubled explanatory power from 27% to 52% of intention variance across 57 studies, while (Klöckner 2013) demonstrated fully integrated models explained 36% of behavioral variance, 55% of intention variance, and 47% of personal norm variance across 56 diverse environmental behaviors—substantially outperforming isolated TPB or NAM applications. (Duong 2024) found that a unified TPB-NAM model provided superior explanatory power for energy-saving behaviors compared to single-theory applications. These gains emerge because integration captures dual pathways: rational cost-benefit calculations (TPB) sustain behavior when moral urgency weakens, while moral obligations (NAM) motivate action when outcomes remain invisible.

For low-carbon behavior specifically, integration shifts from advantageous to imperative. The confluence of invisible outcomes, temporally distant consequences, and documented intention-behavior gaps (Figure 1) creates conditions where neither theoretical pathway functions effectively in isolation: TPB's rational calculations fail without observable feedback; NAM's moral activation weakens across extended timeframes; and both demonstrate limited translation from intention to behavior. Integration addresses this convergence by enabling compensatory mechanisms—when one pathway weakens (e.g., invisible outcomes undermining rational calculation), the other sustains motivation (e.g., moral obligation providing behavioral impetus despite absent feedback). Empirically, (Wang T. et al. 2024) confirmed that low-carbon behaviors require simultaneous engagement of both rational and moral pathways, with integrated models substantially outperforming single-theory approaches.

However, how integration occurs across diverse environmental behaviors remains systematically unmapped. Common integration pathways include: awareness of consequences influencing attitude formation (Chen, 2016), subjective norms activating personal norms through internalization (Thøgersen, 2006), and personal norms shaping attitudes (Klöckner and Blobaum, 2010). Nevertheless, current research demonstrates integration attempts without revealing dominant patterns: which specific TPB-NAM linkages (e.g., awareness → attitude, subjective norm → personal norm) prove most prevalent? Do researchers achieve deep theoretical synthesis or simply combine constructs additively? Which integration pathways optimize explanatory power? By systematically mapping these integration patterns, this study provides evidence-based guidance for constructing theoretically rigorous frameworks particularly suited to behaviors like low-carbon behavior that demand simultaneous engagement of rational and moral pathways.

### 2.4 Conceptual overlap and integration challenges

Integration also raises conceptual concerns that merit explicit attention. Subjective norm (TPB) and personal norm (NAM) both capture normative influences on behavior but operate through distinct mechanisms—perceived external social pressure vs. internalized moral obligation (Ajzen, 1991; Schwartz, 1977)—and a similar conceptual overlap exists between attitude (TPB) and personal norm (NAM), which can both reflect an individual's evaluative stance toward a behavior (Klöckner and Blobaum, 2010). A higher level of explained variance in an integrated model does not, by itself, justify combining TPB and NAM, since explanatory gains may partly reflect construct overlap rather than genuine complementarity (Bamberg and Schmidt, 2003); rigorous integration therefore requires explicit differentiation between overlapping constructs (e.g., discriminant validity testing) rather than reliance on cumulative R^2^ improvements alone. This concern is directly relevant to the pathway analysis reported in Section 5.3, where pathways linking conceptually distinct constructs (e.g., awareness of consequences → attitude) warrant more confidence than those linking constructs with substantial overlap (e.g., subjective norm → personal norm).

## 3 Methods

### 3.1 Research design and data source

**Figure 2:**
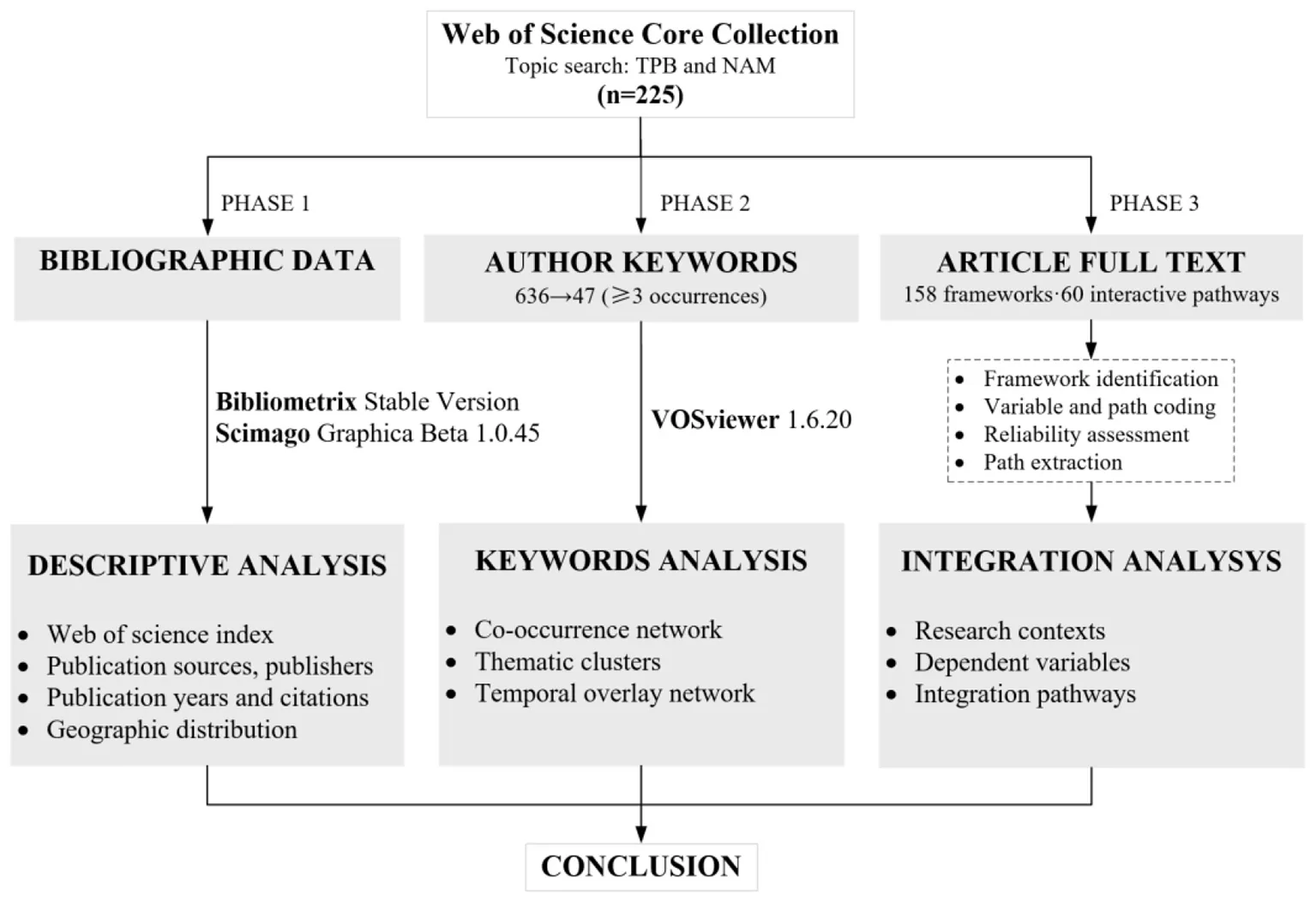
Research procedure.

This study employs a multi-dimensional bibliometric approach combined with systematic content analysis to examine TPB and NAM integration research in environmental behavior. Bibliometric analysis was selected because it provides objective, quantitative measures of research trends, enables visualization of knowledge structures through network analysis, and supports systematic identification of research gaps (Donthu et al., 2021). The research design consists of three analytical phases (Figure 2): Phase 1 conducts a descriptive bibliometric analysis of publication trends, sources, and geographic distribution; Phase 2 performs keyword co-occurrence analysis to identify thematic clusters; and Phase 3 employs systematic content analysis, following the approach of (Kraus et al. 2022), to examine theoretical integration patterns, the full four-step procedure is detailed in Section 3.4. Research data were retrieved on November 16, 2024, from the Web of Science, covering Science Citation Index Expanded (SCIE, since 1970), Social Sciences Citation Index (SSCI, since 1970), Arts & Humanities Citation Index (A&HCI, since 1975), and Emerging Sources Citation Index (ESCI, since 2005). It should be noted that while ESCI is accessible through the Web of Science platform, it applies less stringent selection criteria than the traditional Core Collection indexes (Korman et al., 2024). This single-database approach was a deliberate choice: Web of Science provides the standardized citation and keyword metadata required for the network-based bibliometric techniques used here, and combining it with databases having different metadata structures (e.g., Scopus) risks introducing inconsistencies into such analyses.

### 3.2 Search strategy and sample selection

The search strategy was designed to capture all studies comprehensively integrating TPB and NAM, regardless of specific application domains, since the study's aim is to map how TPB and NAM constructs are theoretically connected in general (Figure 1) rather than to catalog behavior-specific applications, which are examined separately in Section 5.1. The search query was: Topic = (“Theory of Planned Behavior” OR “TPB” OR “Theory of Planned Behavior”) and (“Norm Activation Model” OR “NAM” OR “Norm Activation Theory” OR “NAT” OR “Schwartz^*^ norm^*^”). This broad search approach was intentionally adopted to examine how TPB-NAM integration has been applied across the full spectrum of environmental behavior research, rather than restricting to specific behavioral domains. The resulting sample encompasses various contexts including sustainable consumption, waste management, transportation, energy conservation, and tourism, enabling systematic understanding of integration patterns. It should be clarified that “low-carbon behavior” was not itself used as a search term but functions here as a conceptual umbrella, since low-carbon-relevant actions are typically indexed under broader pro-environmental behavior terminology rather than this specific label. While low-carbon behavior represents an important subset within this broader environmental behavior literature, the domain-agnostic search strategy ensures complete coverage of theoretical integration approaches that can inform low-carbon behavior research specifically.

**Figure 3:**
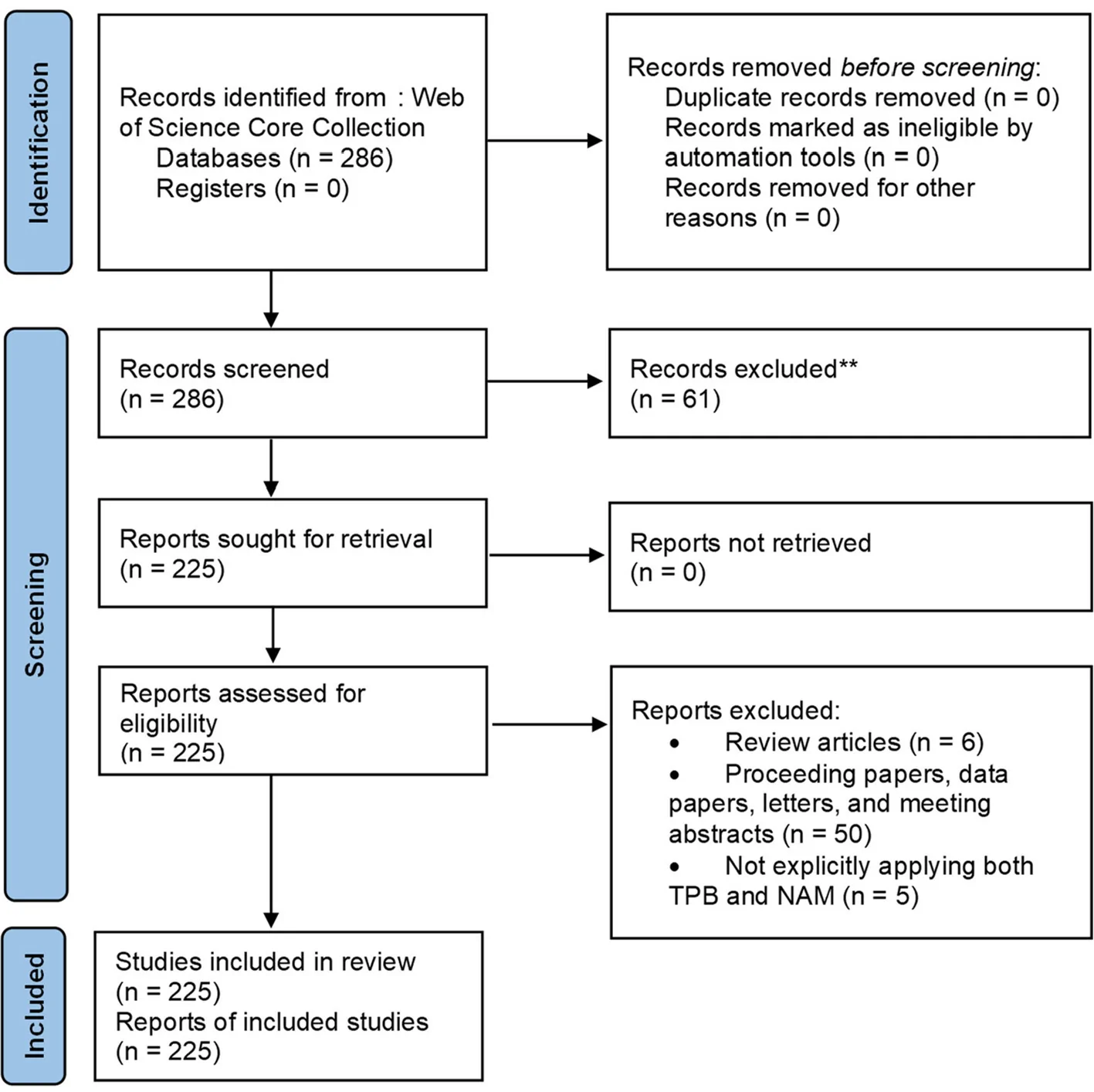
The PRISMA flowchart with literature included/excluded criteria. **Records excluded at the screening stage for not meeting the eligibility criteria specified in Section 3.2 (i.e., peer-reviewed journal articles explicitly applying both TPB and NAM).

Without restrictions on publication years or language, the analysis identified 225 relevant articles for bibliometric analysis after applying inclusion criteria (peer-reviewed journal articles explicitly applying both TPB and NAM) and exclusion criteria (review articles, proceeding papers, data papers, letters, and meeting abstracts) shown in Figure 3 (Abramo et al., 2024; Wei et al., 2024). Although no language restrictions were imposed, all retrieved articles were published in English, reflecting the dominant publication language in this research field. Among the 225 articles, 158 presented explicit research frameworks (conceptual models or hypothesis diagrams) and were further analyzed for integration patterns.

### 3.3 Analytical tools and keyword processing

Three software tools were employed for different analytical purposes. Bibliometrix Stable Version was used for data import and descriptive statistical analysis. VOSviewer (version 1.6.20) was used for data cleaning, constructing, and visualizing keyword co-occurrence networks, with cluster analysis performed using the association strength normalization method and minimum keyword occurrence threshold set at 3. Scimago Graphica (version Beta 1.0.45) was used specifically for geographic visualization of country-level publication distribution and international collaboration networks.

From the 225 articles, 636 author keywords were initially extracted. Keywords were cleaned and standardized through synonym merging (e.g., “TPB” unified to “theory of planned behavior”; “NAM” unified to “norm activation model”), form standardization (singular/plural forms unified; British/American spelling standardized), and threshold application (keywords with fewer than three occurrences excluded). This process was conducted using the VOSviewer thesaurus function with manual verification. The final keyword co-occurrence analysis included 47 keywords meeting the minimum occurrence threshold. The complete keyword correspondence table is provided in Supplementary material, Table A.

### 3.4 Content analysis procedure

To analyze theoretical integration patterns beyond bibliometric mapping, systematic content analysis of full texts was conducted following Kraus et al.'s (2022) guidelines. The procedure consisted of four steps. First, framework identification: two researchers independently reviewed each article to determine whether an explicit research framework was presented, identifying 158 articles (70.2%) with explicit theoretical frameworks. Second, variable and path coding: for the 158 articles with frameworks, we extracted dependent variables (behavioral intention, actual behavior, or both), independent variables (TPB constructs and NAM constructs included), and integration paths (explicit hypothesized relationships connecting TPB and NAM constructs). Third, reliability assessment: inter-coder reliability was assessed on a random sample of 30 articles (19% of articles with frameworks), with Cohen's kappa calculated at 0.85, indicating substantial agreement (Landis and Koch, 1977); disagreements were resolved

through discussion until consensus was reached. Finally, path extraction: from the 158 articles with frameworks, 60 articles (38.0%) proposed explicit interaction paths between TPB and NAM constructs; these paths were extracted, categorized, and quantified to identify dominant integration patterns. Whereas, PRISMA governs the identification, screening, and selection of records (Figure 3), (Kraus et al. 2022) approach was applied specifically to guide the qualitative coding of theoretical constructs and integration pathways from the full texts of included articles, a step not covered by PRISMA's reporting standards.

Throughout this study, the following abbreviations are used: DV, Dependent variable; IV, Independent variable; AT, Attitude; SN, Subjective norm; SoN, Social norm; PBC, Perceived behavioral control; AW, Awareness; AR, Ascription of responsibility; PN, Personal norm(s); INT, Intention; BEH, Behavior.

## 4 Results

This section analyses TPB and NAM integration studies through bibliometric analysis, examining the Web of Science index, top publication sources, publishers, temporal trends and country citations, and keyword co-occurrence analysis to reveal research patterns in this field.

### 4.1 Distribution of Web of Science indexes

Table 2 presents the distribution of articles across Web of Science indexes. Most articles (225, 70.22%) were indexed in the SSCI, indicating this field's primary orientation toward social sciences. A substantial proportion (118, 52.44%) appeared in SCIE, reflecting engagement with natural science journals. Additionally, 24 articles (10.67%) were included in ESCI, and two articles (0.89%) in A&HCI. The cumulative percentage exceeds 100% because individual journals can be indexed in multiple databases simultaneously. This multi-indexing at the journal level reflects the broad disciplinary scope of outlets publishing TPB-NAM integration research, rather than indicating article-level interdisciplinarity.

**Table 2:** Web of science index for TPB + NAM integration.

Web of science index	Record count	% of 225
Social Sciences Citation Index (SSCI)	158	70.22
Science Citation Index Expanded (SCIE)	118	52.44
Emerging Sources Citation Index (ESCI)	24	10.67
Arts and Humanities Citation Index (AandHCI)	2	0.89

### 4.2 Publication sources

The top 12 publication sources for TPB-NAM integration research are shown in Table 3. *Sustainability* leads with 27 articles (12%), followed by *Journal of cleaner production* (eight articles, 3.6%) and *international journal of environmental research and public health* (seven articles, 3.1%). The top 12 journals collectively account for 38.7% of all publications. Notably, high-impact journals in environmental psychology (*Journal of Environmental Psychology*, JIF = 6.1) and resource management (*Resources, conservation, and recycling*, JIF = 11.2) feature prominently, indicating recognition of TPB-NAM integration research within established disciplinary outlets. The presence of transportation and hospitality management journals reflects the diverse application domains of this theoretical integration.

**Table 3:** Top 12 publication sources for TPB-NAM integration research.

No.	Journal	Articles	% of 225	JIF2023	Publisher
1	Sustainability	27	12.0%	3.3	MDPI
2	Journal of Cleaner Production	8	3.6%	9.7	Elsevier
3	International Journal of Environmental Research and Public Health	7	3.1%	–	MDPI
4	Environment, Development, and Sustainability	6	2.7%	4.7	Springer
5	Resources, Conservation, and Recycling	6	2.7%	11.2	Elsevier
6	Transportation Research Part F: Traffic Psychology and Behavior	6	2.7%	3.5	Elsevier
7	Frontiers in Psychology	5	2.2%	2.6	Frontiers
8	International Journal of Hospitality Management	5	2.2%	10.0	Elsevier
9	Journal of Environmental Psychology	5	2.2%	6.1	Elsevier
10	Heliyon	4	1.8%	3.4	Cell press
11	Journal of Sustainability Tourism	4	1.8%	6.9	Taylor and Francis
12	Management of environmental quality	4	1.8%	4.6	Emerald

JIF, Journal Impact Factor (2023). International Journal of Environmental Research and Public Health was indexed in JCR up to the 2021 edition (4.614) but was removed from the Web of Science Core Collection in 2023 and therefore no longer receives a JCR Impact Factor thereafter.

### 4.3 Publishers and publication trends

**Figure 4:**
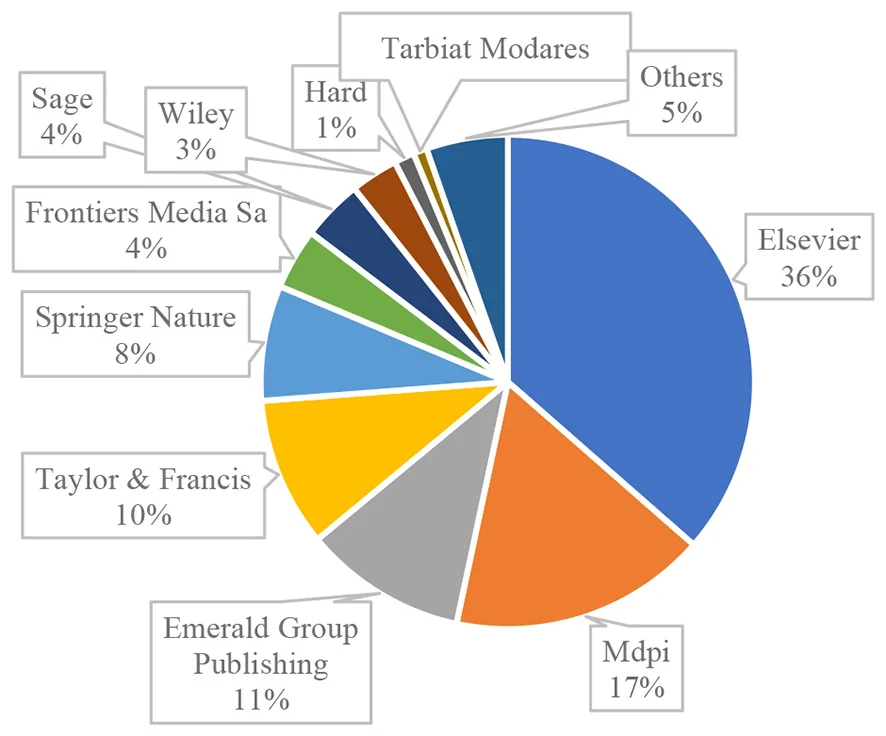
Publishers for TPB + NAM integration research.

Figure 4 shows the distribution of publishers for TPB + NAM integration research. Elsevier ranks first with 36%, followed by MDPI (17%), Emerald Group Publishing (11%), Taylor & Francis (10%), and Springer Nature (8%). The concentration of publications in these major academic publishers—with the top three collectively accounting for 64%—reflects the established academic status of TPB-NAM integration research.

**Figure 5:**
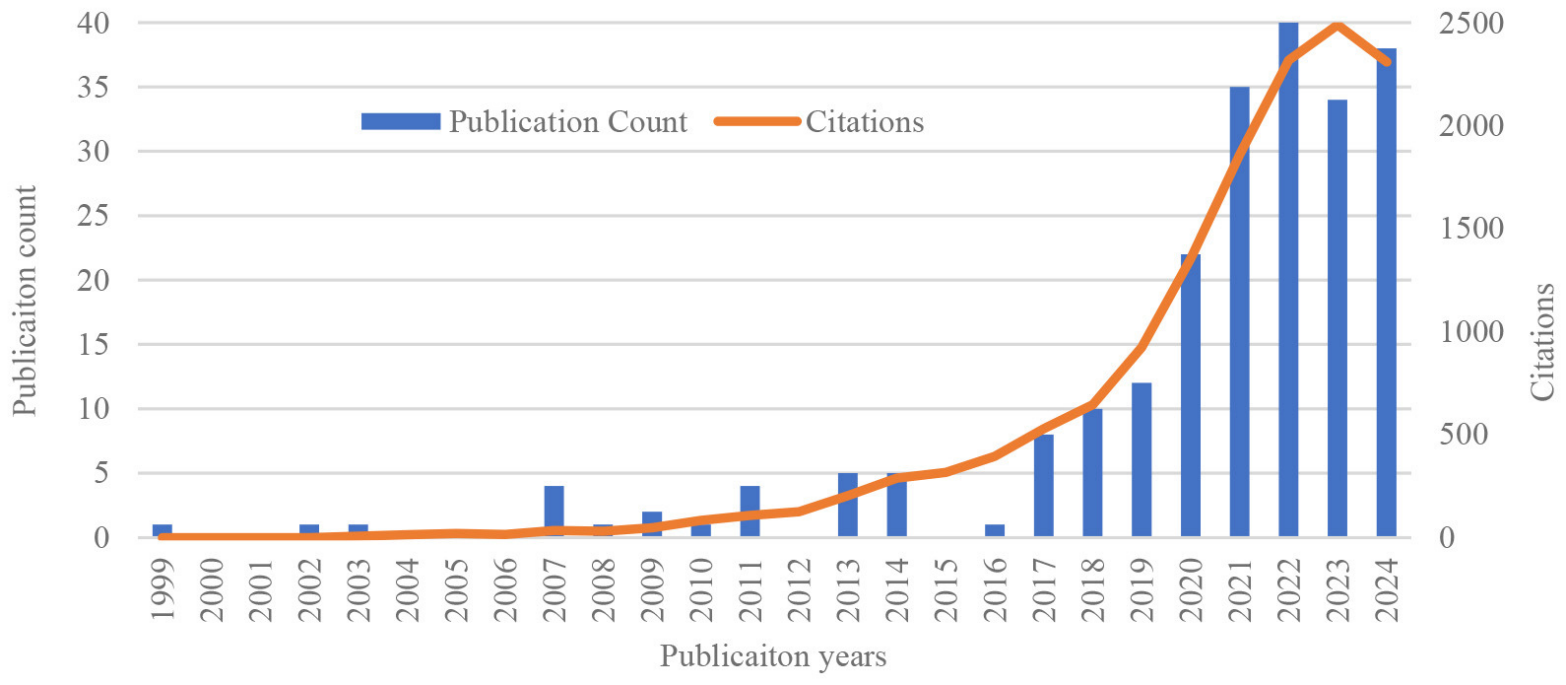
Publication years and citations for TPB + NAM integration research.

Figure 5 shows the publication volume and citation trends from 1999 to 2024. No related research published before 1999. The field remained relatively dormant during 1999–2016, with annual publications generally below five articles. A noticeable increase began in 2017, accelerating sharply from 2020 and peaking in 2022 with 40 publications. This growth trajectory coincides with increasing global attention to climate change and sustainable development, suggesting that TPB-NAM integration has gained recognition as a valuable framework for understanding environmental behavior. The citation trend shows exponential growth, reaching 2,490 citations in 2023, indicating the field's growing academic influence.

### 4.4 Geographic distribution

**Figure 6:**
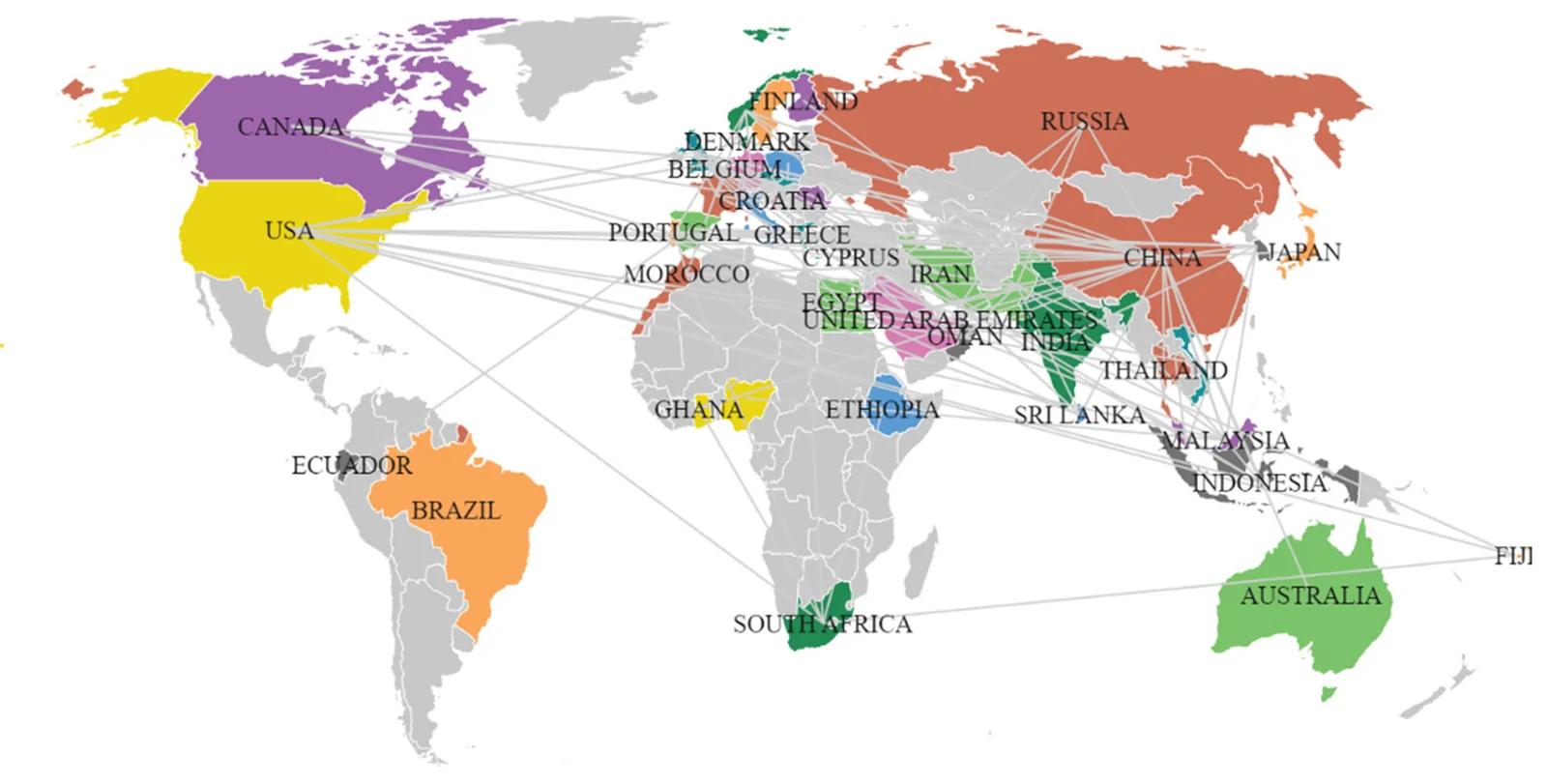
The international collaboration network.

Table 4 and Figure 6 present the geographic distribution of 225 publications on TPB-NAM integration research. Among the 48 countries, China dominates the field with 99 publications (44.0%), followed by the United Kingdom (25, 11.1%) and South Korea (23, 10.2%). The concentration in Asian countries (China, South Korea, Malaysia, Vietnam, and Indonesia collectively representing over 70% of publications) suggests strong regional interest in applying integrated theoretical frameworks to environmental behavior research. The international collaboration network visualized in Figure 6 reveals that China serves as a central hub connecting Asian and Western research communities. This geographic pattern may reflect both the urgency of environmental challenges in rapidly developing Asian economies and the growing research capacity in these regions.

**Table 4:** Top 10 countries contributing to TPB-NAM integration research.

No.	Country	Articles	% of 225
1	China	99	44.0%
2	United Kingdom	25	11.1%
3	South Korea	23	10.2%
4	Malaysia	20	8.9%
5	United States	20	8.9%
6	Vietnam	15	6.7%
7	Germany	12	5.3%
8	India	10	4.4%
9	Netherlands	10	4.4%
10	Saudi Arabia	8	3.6%

Percentages based on 225 total articles. Single articles may involve multiple countries through international collaboration.

### 4.5 Keyword co-occurrence analysis

**Figure 7:**
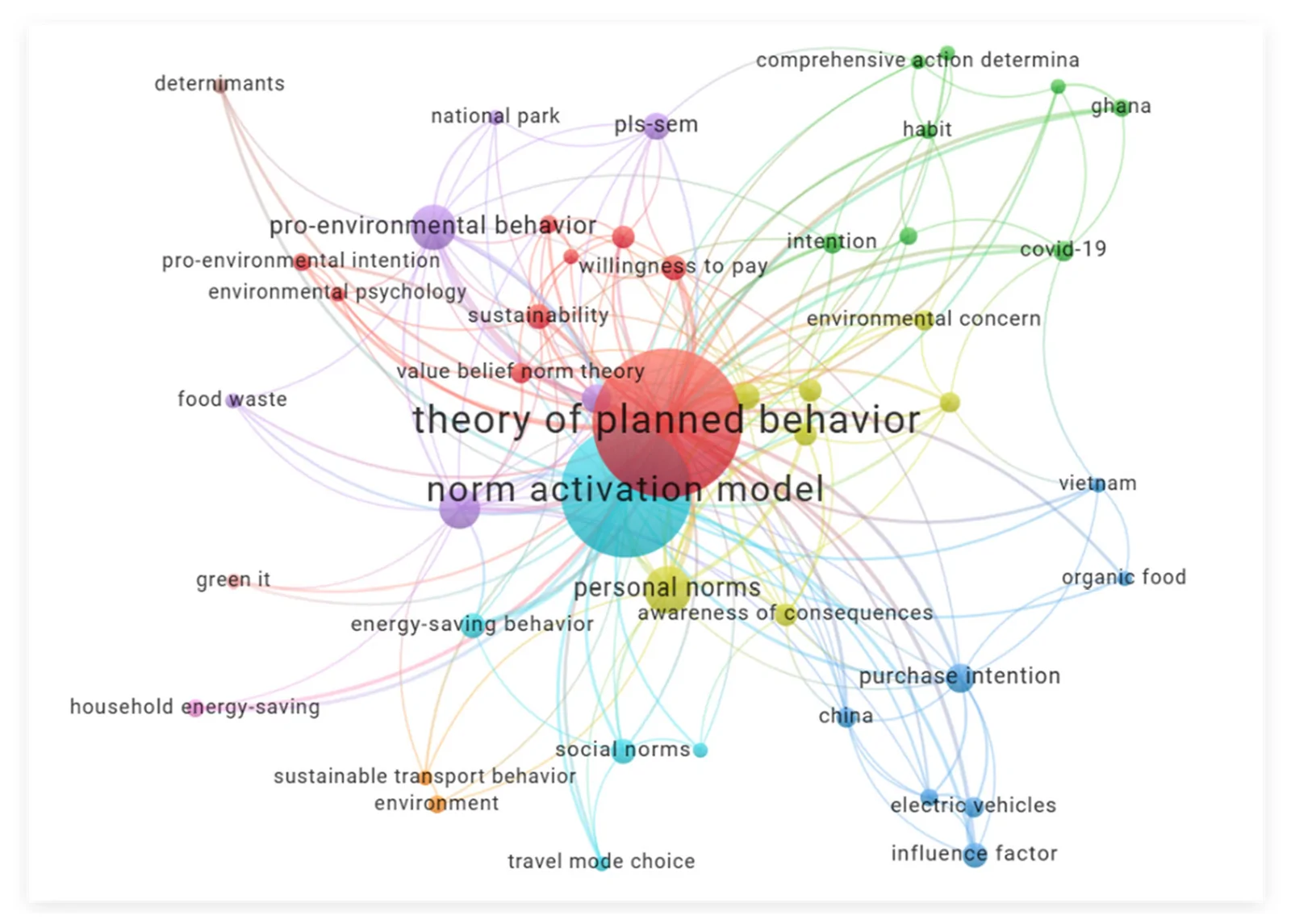
Keyword co-occurrence network for TPB + NAM integration research.

From 636 keywords in the 225 articles, 47 keywords meeting the minimum occurrence threshold of three were selected for co-occurrence analysis. The keyword co-occurrence network (Figure 7) reveals six distinct clusters representing the intellectual structure of TPB-NAM integration research:

1. Red cluster: theory of planned behavior and psychological determinants.
2. Cyan cluster: norm activation model and normative influences.
3. Purple cluster: pro-environmental behavior, methodological approaches, and practical applications.
4. Green cluster: behavioral routines and waste practices.
5. Blue cluster: integrated framework and electric vehicles.
6. Yellow cluster: norm and awareness.

These clusters reveal meaningful patterns in the field's knowledge structure. The red and cyan clusters represent the two theoretical pillars, with red capturing TPB's core constructs (attitude, subjective norm, and perceived behavioral control) and cyan capturing NAM's normative components (personal norm and awareness of consequences). The separation and proximity of the two clusters indicate that while the theories maintain distinct identities, researchers frequently bridge them in empirical applications. The purple cluster functions as a methodological hub, with “structural equation modeling” reflecting this method's dominance in testing integrated frameworks. The green cluster (waste separation, recycling) and blue cluster (electric vehicles, China) represent mature and emerging application domains, respectively. The yellow cluster captures bridging constructs—“norm,” “awareness,” and “moral norm”—that theoretically connect TPB and NAM. The prominence of awareness, norm, and moral norm within the network suggests that the field has increasingly shifted from examining isolated theoretical constructs toward understanding the psychological mechanisms linking cognitive evaluations, social influence, and moral regulation. This trend provides empirical support for integrated perspectives on environmental behavior and highlights the growing relevance of TPB–NAM integration within environmental psychology.

**Figure 8:**
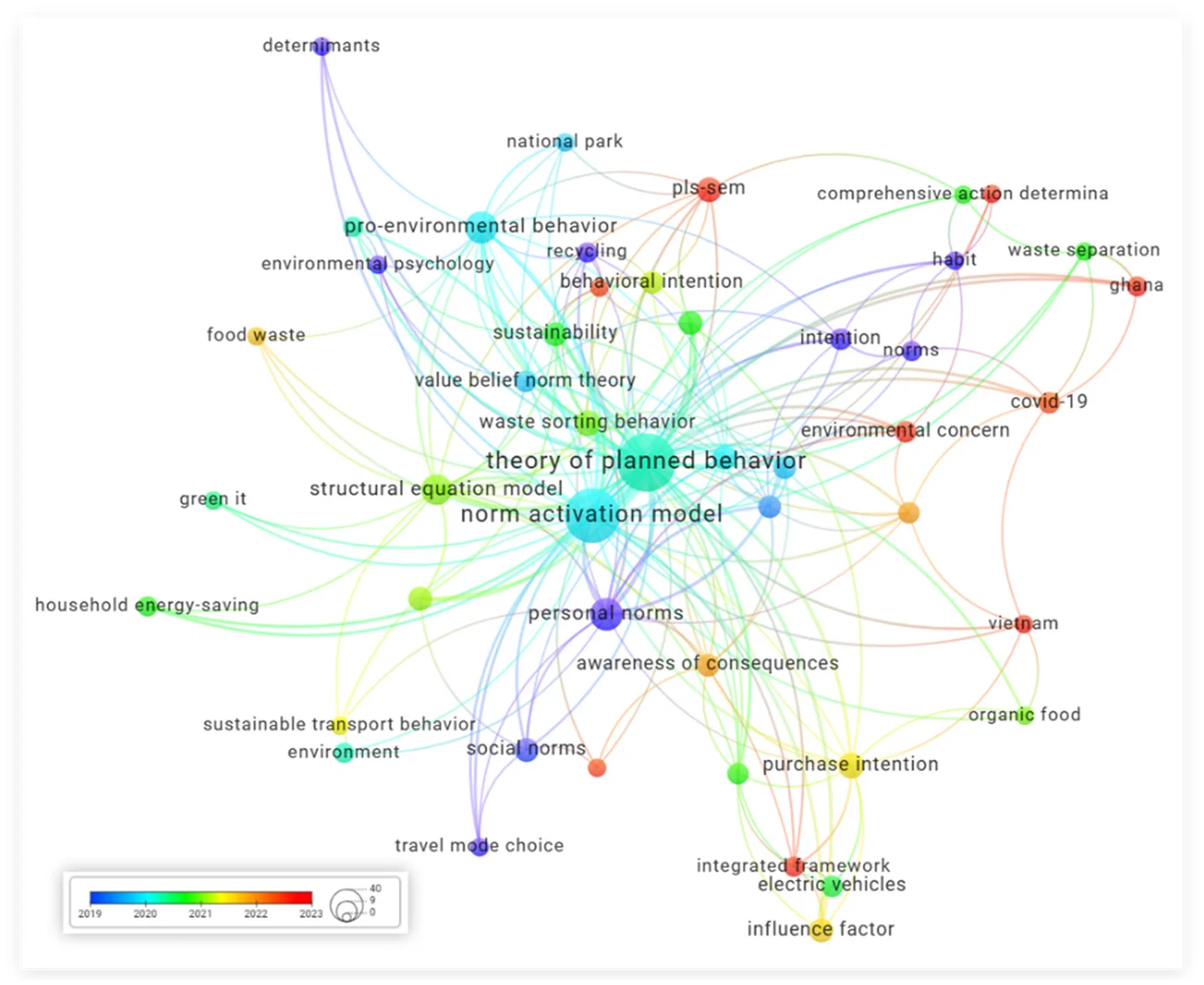
Keyword co-occurrence in overlay network for TPB + NAM integration research.

The temporal overlay network (Figure 8) illustrates the field's evolution: early studies (2019–2020) focused on foundational theoretical constructs; mid-period research (2021) expanded into specific domains like waste separation and sustainable transport; while recent research (2022–2023) shows increased attention to developing countries (Vietnam, Ghana), methodological innovation through PLS-SEM, and emerging topics including COVID-19 impacts.

## 5 Discussion

### 5.1 Research contexts in integrating TPB and NAM

Among the 158 articles with explicit frameworks, TPB-NAM integration research predominantly addresses environmental and sustainable development behaviors (Table 5). Five major domains account for 82.3% of studies: sustainable consumption (24.1%), waste management (17.7%), sustainable transportation (15.2%), tourism and hotels (15.2%), and agriculture and production (10.1%). Key subcategories include green product purchasing (22 articles), household waste sorting and recycling (19 articles), energy conservation and resource saving (16 articles), electric/new energy vehicles (14 articles), and tourism (14 articles). Additional research spans sustainable travel modes, environmental governance, and enterprise production, demonstrating the framework's versatility across both individual and organizational levels. Notably, these domains predominantly address behaviors with direct or indirect carbon reduction implications—from consumption choices affecting carbon footprints to transportation and energy conservation—suggesting that TPB-NAM integration has organically gravitated toward behaviors central to climate change mitigation.

**Table 5:** Research contexts in integrating TPB and NAM.

First-level category	Second-level category	References
Sustainable consumption (38)	Green product purchasing (22)	Alzubaidi et al., 2021; Asmi et al., 2022; Borozan and Pfeifer, 2023; Borusiak et al., 2020, 2022; Dutta et al., 2022; Fabiana et al., 2024; He and Sui, 2024; Jiang et al., 2024; Le and Nguyen, 2022; Liu et al., 2024; Nguyen, 2022; Phu et al., 2024; Richter and Hunecke, 2020; Severijns et al., 2023; Shin et al., 2018; Tverskoi et al., 2021; Wang C. et al., 2024; Xu Y. et al., 2024; Zerbini et al., 2019; Zhu et al., 2024; Zur and Klöckner, 2014
	Energy conservation and resource saving (16)	Bhutto et al., 2021; Fu et al., 2021; Ji and Chan, 2019; Jia et al., 2024; Li et al., 2019, 2020; Nketiah et al., 2022; Palani and Karatas, 2021; Tverskoi et al., 2021; Wang B. et al., 2018; Wang and Lin, 2024; Wang et al., 2019; Xu et al., 2021; Xuan et al., 2023; Zhao et al., 2024
Waste management (28)	Household waste sorting and recycling (19)	Ding et al., 2023; Esfandiar et al., 2020, 2021; Goh et al., 2022; Liu et al., 2022; Muthukumari et al., 2024; Ofstad et al., 2017; Prabhu and Majhi, 2024; Setiawan et al., 2021; Shen et al., 2020, 2022; Shi et al., 2021; Sonnenberg et al., 2022; Wan et al., 2014; Wang Z. et al., 2018; Xia et al., 2021; Zhang et al., 2019, 2020; Zhang S. et al., 2024
	Food waste management (9)	Heidig et al., 2024; Obuobi et al., 2022; Rastegari et al., 2023; Setiawan et al., 2024; Teng et al., 2022; T'ing et al., 2021; Wang A. et al., 2024; Yu et al., 2021; Zheng et al., 2023
Sustainable transportation (24)	Electric/new energy vehicles (14)	Asadi et al., 2021; Dong et al., 2020; Du et al., 2018; Hamzah and Tanwir, 2021; Ji et al., 2024; Kastner et al., 2021; Klöckner et al., 2013; Li et al., 2024; Lv et al., 2024; Peters et al., 2011; Srivastava et al., 2023; Wall et al., 2008; Wang et al., 2022; Zhang et al., 2018
	Sustainable travel modes (10)	Abdoulaye et al., 2024; Bachmann et al., 2018; Bamberg and Schmidt, 2003; Cui et al., 2025; Davison et al., 2014; Klöckner and Blobaum, 2010; Mosca et al., 2024; Nguyen-Phuoc et al., 2025; Olsson et al., 2018; Sheng and Zhang, 2022
Tourism and hotels (24)	Tourism (14)	(Bai and Zhang, 2021; Han et al., 2018; Han and Hyun, 2017; Jeon et al., 2022; Li and Wu, 2019; Liu J. et al., 2020; Manosuthi et al., 2020; Meng et al., 2020; Ong and Musa, 2011; Sun et al., 2022; Wang et al., 2021; Woosnam et al., 2023; Zhang et al., 2022; Zhu et al., 2022)
	Hotel and catering services (10)	Choe et al., 2020; Chuah et al., 2022; Eid et al., 2021; Joo et al., 2022; Kim and Hwang, 2020; Kim, 2023; Leong and Koay, 2023; Meng et al., 2024; Prisco et al., 2025; Saleem, 2021
Agriculture and production (16)	Agricultural production behavior (10)	Chen et al., 2024; Coon et al., 2020; Coulibaly et al., 2021; Lou et al., 2021; Rezaei et al., 2019; Savari et al., 2023; Wang J. et al., 2023; Wang X. et al., 2023; Xu Z. et al., 2024; Zhao et al., 2024
	Enterprise production behavior (6)	Asadi et al., 2019; Dada et al., 2024; Liu Q. et al., 2020; Meng et al., 2022; Nascimento Lopes et al., 2019; Zhao et al., 2023
Environmental governance and participation (15)	Environmental governance participation (10)	Arkorful, 2022; Borozan and Pfeifer, 2023; Ma et al., 2022; Shi et al., 2017, 2022; Yang et al., 2022; Zhang et al., 2017, 2023; Zhang and Wang, 2022; Zhao et al., 2016
	Community and housing (5)	Cheng et al., 2022; Rissetto et al., 2022; Sang et al., 2020; Xiao et al., 2023; Zhou et al., 2023
Theoretical and methodological research (7)	Theoretical and methodological research (7)	Bamberg, 2013; Concari et al., 2023; Kim et al., 2018; Klöckner, 2013; Onwezen et al., 2013; Trihadmojo et al., 2020; Wall et al., 2008
Other social behavior (6)	Other social behavior (6)	Almokdad et al., 2023; Chen, 2020; Chen et al., 2019; Liu Q. et al., 2020; van Kesteren et al., 2007; Verma et al., 2020

### 5.2 Dependent variables in TPB and NAM integration studies

Analysis of the 158 articles with explicit research frameworks reveals behavioral intention as the predominant dependent variable: 135 articles (85.4%) exclusively examined behavioral intention, 17 investigated both behavioral intention and behavior (Table 6), and only six articles focused solely on actual behavior (Table 7). This emphasis on intention is particularly problematic for low-carbon behavior research, where the intention-behavior gap is pronounced due to the invisible, long-term nature of carbon reduction outcomes.

**Table 6:** Studies combining TPB and NAM examined both intention and behavior.

No.	Research contexts	Articles
1	Environmental protection	Borozan and Pfeifer, 2023; Obuobi et al., 2022; Onwezen et al., 2013; Setiawan et al., 2021; Xu Z. et al., 2024; Zhang et al., 2019, 2020; Zhang S. et al., 2024
2	Energy conservation	Fu et al., 2021; Jia et al., 2024; Li et al., 2020; Xuan et al., 2023
3	Transportation	Mosca et al., 2024; Sheng and Zhang, 2022
4	Others	Klöckner, 2013; Yu et al., 2021; Zhao et al., 2016

**Table 7:** Articles with behavior as the only DV jointly applied TPB and NAM.

No.	References	DV	IV
1	(Ong and Musa 2011)	Underwater behavior	Attitude, social norm, and personal norm
2	(Peters et al. 2011)	Vehicle CO_2_ emissions (fuel consumption behavior)	Valence of less power and size, social norm, personal norm, and response efficacy
3	(Esfandiar et al. 2020)	Pro-environmental binning behavior	Attitude, social norm, and personal norm
4	(Esfandiar et al. 2021)	Binning behavior	Attitude, social norm, and personal norm
5	(Goh et al. 2022)	Waste separation behavior	Attitude, social norm, personal norm, and government support
6	(Heidig et al. 2024)	Food waste behavior	Motivation (awareness, attitude, personal norm), opportunity (infrastructure, social norm), and ability (knowledge, skills)

Among the 17 studies examining both variables, research spans environmental protection, including waste sorting behavior (Setiawan et al., 2021; Zhang et al., 2019, 2020; Zhang S. et al., 2024), and low-carbon behavior (Borozan and Pfeifer, 2023), energy conservation primarily focusing on household electricity-saving behavior (Fu et al., 2021; Li et al., 2020) and energy-efficient appliance purchasing decisions (Jia et al., 2024), and transportation exploring public transit use (Sheng and Zhang, 2022) and sustainable commuting (Mosca et al., 2024). Notably, 15 of these 17 studies employed cross-sectional designs with simultaneous measurement, contradicting TPB's fundamental premise that intention temporally precedes behavior (Ajzen, 1991). Exceptions such as (Mosca et al. 2024) and Klöckner [20] demonstrated more rigorous methodologies through longitudinal design and meta-analysis, respectively. Especially, among the six articles that directly studied behavior without intention (Table 7), some researchers justified this choice by citing the significant intention-behavior gap and the higher reliability of current behavioral data compared to future intentions—a rationale particularly relevant for mature, established behaviors (Esfandiar et al., 2020, 2021; Goh et al., 2022). Future research should prioritize studying actual behavior measurement with appropriate temporal designs, especially in low-carbon contexts where bridging the intention-behavior gap is critical.

### 5.3 Integration approaches of TPB and NAM

**Figure 9:**
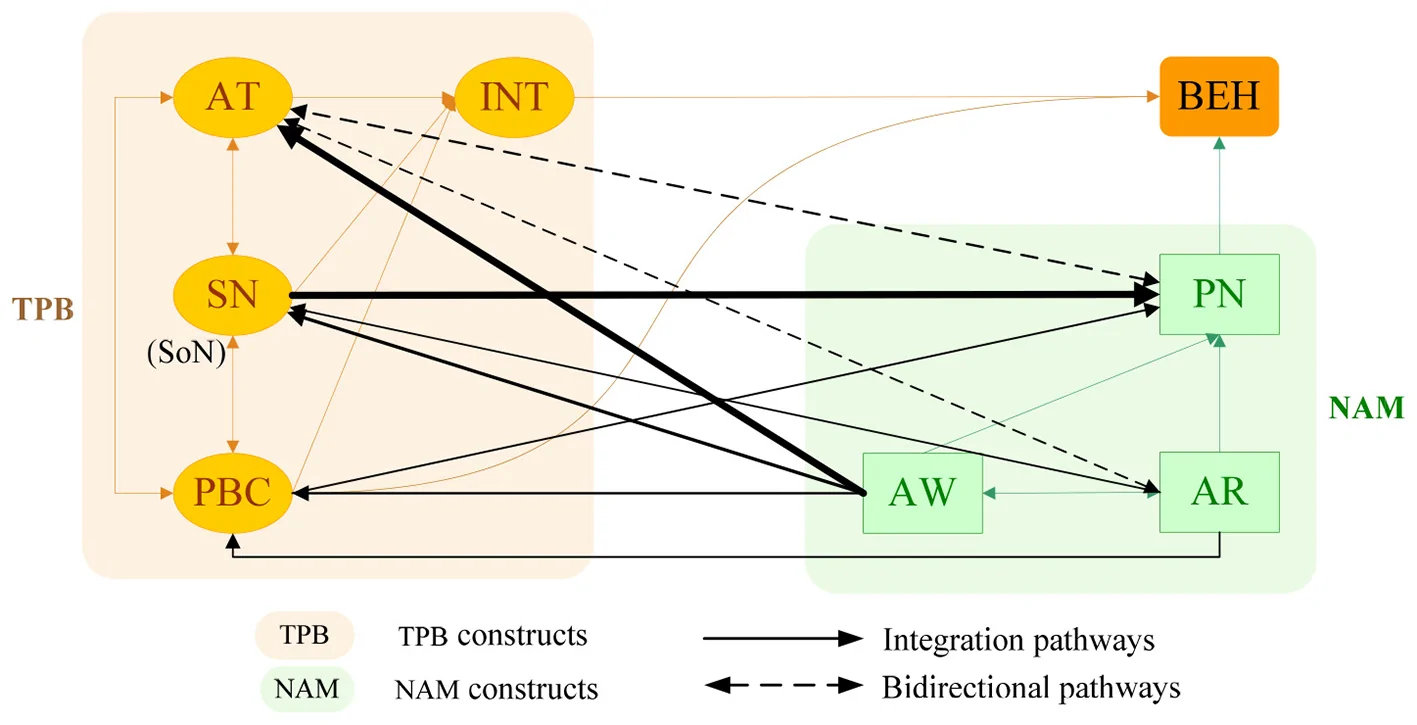
Path analysis of TPB and NAM integration studies. BEH, Behavior; PN, Personal norm(s); AW, Awareness; AR, Ascription of responsibility; AT, Attitude; SN, Subjective norm(s); SoN, Social norm(s); PBC, Perceived behavioral control; INT, Intention.

Among the 158 articles, 60 studies (38.0%) proposed specific integration pathways connecting TPB and NAM constructs (Table 8), while 98 studies (62.0%) applied both theories without explicit construct interactions (Table 9). From these 60 interactive studies, 129 pathway instances across 13 distinct path types were identified (Table 10, Figure 9). Four dominant pathways account for 78.3% of all integration attempts: awareness → attitude (AW → AT, 31.8%), subjective norm → personal norm (SN → PN, 23.3%), awareness → subjective norm (AW → SN, 14.7%), and social norm → personal norm (SoN → PN, 8.5%). The prominence of awareness as a starting point for multiple pathways highlights its crucial bridging role—consequence awareness not only activates personal norms as proposed by NAM but also shapes attitudes and perceived social expectations linking to TPB. Significant bidirectional relationships were identified between attitude and personal norm (PN → AT, 7 instances; AT → PN, 4 instances) and between attitude and ascription of responsibility (AR → AT, 2 instances; AT → AR, 1 instance), indicating complex interplay rather than simple unidirectional influence. These patterns reveal researchers' focus on specific influence paths while highlighting the importance of examining bidirectional relationships in behavioral formation.

**Table 8:** Summary of articles on construct interaction research between TPB and NAM.

No.	Interaction paths	Interaction paths	Articles
1	5	AW → AT, AW → SN, AR → AT, AR → SN, SN → PN	Arkorful et al., 2023
2	4	AW → AT, AW → PBC, SN → PN, SN → AR	Wang X. et al., 2023
3	4	AW → AT, AW → SN, AR → PBC, SN → PN	Xu Z. et al., 2024
4	4	AW → AT, AW → SN, AT → PN, SN → PN	Zhou et al., 2023
5	4	AW → AT, AW → SN, AW → PBC, SN → PN	Liu J. et al., 2020; Sheng and Zhang, 2022
6	4	AW → AT, AW → SoN, SoN → AR, SoN → PN	Shen et al., 2020
7	4	AR → AT, AR → SoN, PBC → AR, SoN → PN	Onwezen et al., 2013
8	3	AT → PN, PBC → PN, SoN → PN	Severijns et al., 2023
9	3	AW → AT, AW → SN, AW → PBC	Almokdad et al., 2023; Kim et al., 2018; Meng et al., 2020; Nketiah et al., 2022
10	3	AW → AT, AW → SN, SN → PN	Arkorful, 2022; Ding et al., 2023; Rezaei et al., 2019; Sun et al., 2022; Zhang et al., 2017; Zhao et al., 2016
11	3	AW → AT, PN → AT, SN → PN	Zhang and Wang, 2022
12	3	PN → SoN, PN → AT, PN → PBC	Cui et al., 2025
13	2	AR → PBC, PN → PBC	Jiang et al., 2024
14	2	AT → PN, SN → PN	van Kesteren et al., 2007
15	2	AT → PN, SoN → PN	Coon et al., 2020
16	2	AW → AT, AR → AT	Zhang et al., 2019
17	2	AW → AT, AT → AR	Rastegari et al., 2023
18	2	AW → AT, AW → SN	Nguyen, 2022; Trihadmojo et al., 2020; Wang J. et al., 2023
19	2	AW → AT, PN → AT	Mosca et al., 2024
20	2	AW → AT, SN → PN	Coulibaly et al., 2021; Han and Hyun, 2017; He and Sui, 2024; Ji et al., 2024; Joo et al., 2022; Kim and Hwang, 2020; Kopaei et al., 2021; Le and Nguyen, 2022; Liu et al., 2024; Savari et al., 2023; Zhang S. et al., 2024
21	2	AW → AT, SoN → PN	Esfandiar et al., 2020, 2021
22	2	AW → AT, AW → SN	Chen, 2020
23	2	AW → SoN, SoN → PN	Peters et al., 2011; Sonnenberg et al., 2022
24	2	SN → PN, PBC → PN	Xiao et al., 2023
25	2	SoN → PN, PBC → PN	Klöckner, 2013
26	1	AW → AT	Jia et al., 2024; Woosnam et al., 2023
27	1	PN → AT	Dada et al., 2024; Prisco et al., 2025; Wang et al., 2022; Zerbini et al., 2019
28	1	SN → PN	Han et al., 2017; Ong and Musa, 2011; Wang Z. et al., 2018; Zhu et al., 2024
29	1	SoN → PN	Chen et al., 2024; Goh et al., 2022

AW, Awareness; AT, Attitude; AR, Ascription of responsibility; PBC, Perceived behavioral control; PN, Personal norm(s); SN, Subjective norm(s); SoN, Social norm(s).

**Table 9:** Integration pathway number between TPB and NAM studies.

Interaction paths per article	Number of articles	Percentage of total articles
5	1	0.63%
4	7	4.43%
3	13	8.23%
2	27	17.09%
1	12	7.59%
0	98	62.03%
Total	158		

**Table 10:** Path analysis of joint studies on TPB and NAM.

Interaction paths	Frequency	Percentage of total interaction paths
AW → AT	41	31.8%
SN → PN	30	23.3%
AW → SN	19	14.7%
SoN → PN	11	8.5%
AW → PBC	7	5.4%
PN → AT	7	5.4%
AT → PN	4	3.1%
PBC → PN	3	2.3%
AR → AT	2	1.6%
AR → PBC	2	1.6%
AT → AR	1	0.8%
AR → SoN	1	0.8%
PBC → AR	1	0.8%
Total	129	

However, the vigorous development of these integration studies has also led to a series of issues, such as arbitrariness in integration methods and weak theoretical foundations. For example, (Wang B. et al. 2018) highly cited study proposed relationships between attitude and NAM variables by citing (Chen 2016), yet Chen's research focused on moral obligation as a TPB extension and did not examine these relationships. Similarly, while citing TPB-based studies that examined behavioral intentions, (Wang B. et al. 2018) directly hypothesized attitude-behavior relationships without explaining the omission of the intention construct central to TPB theory. Such issues highlight the need for more rigorous theoretical integration approaches that respect the internal logic of both theories.

### 5.4 Implications for environmental psychology

The findings of this review provide important implications for environmental psychology by demonstrating that environmental behavior is shaped by the interaction of cognitive, social, and moral processes rather than by a single psychological mechanism. Consistent with the complementary strengths of TPB and NAM, rational evaluations of attitudes, subjective norms, and perceived behavioral control (Ajzen, 1985) operate alongside moral motivations derived from awareness of consequences and personal norms (Schwartz, 1975). The dominance of awareness-related pathways (AW → AT and AW → SN) identified in this review further suggests that awareness functions as a critical psychological bridge linking moral cognition with rational decision-making. This observation supports previous arguments that integrated models offer stronger explanatory power than either TPB or NAM alone (Bamberg and Möeser, 2007; Klöckner, 2013).

The review also highlights the importance of norm internalization in environmental behavior formation. The frequent occurrence of subjective norm → personal norm and social norm → personal norm pathways indicates that external social expectations may gradually develop into internalized moral commitments, providing a psychological explanation for how environmental actions become self-regulated behavior. Furthermore, the predominance of behavioral intention as the dependent variable and the limited attention to actual behavior reinforce concerns regarding the intention–behavior gap in environmental research (Bamberg and Möeser, 2007; Steg and Vlek, 2009). This issue is particularly relevant to low-carbon behavior, where outcomes are often invisible and delayed. Collectively, these findings suggest that future environmental psychology research should move beyond single-theory explanations and further explore how cognitive evaluation, social influence, and moral regulation jointly contribute to the translation of environmental concern into actual behavior.

## 6 Conclusion

This systematic analysis of 225 articles reveals the development trajectory and knowledge structure of TPB-NAM integration research in environmental behavior. The field has experienced rapid growth since 2019, with studies primarily addressing sustainable consumption (24.1%), waste management (17.7%), sustainable transportation (15.2%), and tourism (15.2%)—domains with direct implications for carbon reduction and climate change mitigation. Among the 60 studies proposing explicit construct interactions, four dominant integration pathways emerged: awareness → attitude (31.8%), subjective norm → personal norm (23.3%), awareness → subjective norm (14.7%), and social norm → personal norm (8.5%), collectively accounting for 78.3% of all integration attempts. However, critical limitations persist: most studies (62.03%) employ simple theoretical combinations without deep integration; behavioral intention dominates as the dependent variable (85.4%), while actual behavior measurement remains limited; cross-sectional designs prevail even when examining intention-behavior relationships; and issues of citation-hypothesis disconnects and inconsistent construct operationalization undermine the theoretical rigor of many integration attempts.

This study contributes to the literature by providing systematic mapping of TPB-NAM integration research across 25 years, critically analyzing integration quality issues that have constrained theoretical advancement, and articulating why low-carbon behavior's unique characteristics—long-term orientation, invisible outcomes, and pronounced intention-behavior gaps—make rigorous TPB-NAM integration especially valuable. Future research should address the identified gaps through longitudinal designs examining TPB-NAM construct interactions over time, meta-analytic comparison of effect sizes across integration pathways, and development of domain-specific frameworks tailored to low-carbon behavior. Researchers are advised to prioritize theoretically justified pathways (particularly AW → AT and SN → PN), measure actual behavior rather than solely intentions, and carefully distinguish among different norm constructs. For practitioners designing environmental interventions, the prominence of awareness as a bridging construct suggests that combining consequence awareness with both attitudinal and normative messaging may be more effective than single-pathway approaches.

For climate change policy-makers and practitioners designing behavioral interventions, our findings offer several strategic implications. First, the prominence of awareness as a bridging construct suggests that combining consequence awareness with both attitudinal and normative messaging may be more effective than single-pathway approaches. Second, given that 85.4% of studies measure only behavioral intentions rather than actual behaviors, policy evaluations should incorporate long-term behavioral tracking to assess genuine climate action outcomes. Third, the identified intention-behavior gap in low-carbon contexts indicates that interventions should move beyond awareness campaigns to address implementation barriers through structural and contextual support mechanisms. These insights can inform the design of more effective climate change communication strategies and carbon reduction policies.

This study is limited by its reliance on Web of Science, potentially excluding relevant research from other regional databases such as Scopus or CNKI. Future reviews could incorporate multiple databases for more systematic coverage.

## Data Availability

The raw data supporting the conclusions of this article will be made available by the authors, without undue reservation.
