# Supplementary material for: Rational decisions or moral obligations? A systematic review of TPB-NAM integration in understanding low-carbon behavior
Source: Front Psychol. 2026 Aug 18;17:1894328. doi: 10.3389/fpsyg.2026.1894328 (PMC13529495; doi:10.3389/fpsyg.2026.1894328)
Supplement: Supplementary file 1 [file Table_1.docx]

**Supplementary Material**

**Table A**. **Keywords Processing and Cleaning.**

| **Keywords** | **Replace by** |
| --- | --- |
| attitudes | attitude |
| awareness of consequence | awareness of consequences |
| behavioral intentions | behavioral intention |
| behavioural intention | behavioral intention |
| behavioural change | behavioral change |
| citizens' participation | citizen participation |
| comprehensive action determination modelling | comprehensive action determination model |
| comprehensive action determination models | comprehensive action determination model |
| comprehensive activation determination model | comprehensive action determination model |
| consumers' behaviour | consumer behavior |
| consumers' intentions | consumer behavioral intention |
| consumer intentions | consumer intention |
| consumption intention | consumer intention |
| determinant | deternimants |
| ecological behaviour | ecological behavior |
| electric cars | electric vehicles |
| energy conversion | energy saving behavior |
| energy saving | energy-saving behavior |
| energy -saving behavior | energy-saving behavior |
| energy-saving behaviors | energy-saving behavior |
| energy-saving behaviour | energy-saving behavior |
| energy-saving behaviours | energy-saving behavior |
| energy -efficient household appliances | energy-efficient appliances |
| environmental concerns | environmental concern |
| environment concern | environmental concern |
| extended theory of planned behaviour model | extended theory of planned behavior |
| food waste prevention | food-saving behavior |
| green-it | green it |
| green purchasing behavior | green purchase behavior |
| household energy saving | household energy-saving |
| household electricity-saving | household energy-saving |
| household energy-saving behaviors | household energy-saving |
| household energy-saving option | household energy-saving |
| household reserve efficiency | household energy-saving |
| influencing factor | influence factor |
| influencing factors | influence factor |
| influential factors | influence factor |
| influence factors | influence factor |
| integrated model | integrated framework |
| integrating model | integrated framework |
| integrative theory | integrated framework |
| integrative tpb-nam mode | integrated model of tpb-nam |
| intentions | intention |
| meta-analysis structural equation modeling (masem) | meta-analysis structural equation modeling |
| meta-analytic sem | meta-analysis structural equation modeling |
| meta-analytic structural equation modeling | meta-analysis structural equation modeling |
| nam | norm activation model |
| national parks | national park |
| norm activation | norm activation model |
| norm activation mode | norm activation model |
| norm-activation model | norm activation model |
| norm-activation-model | norm activation model |
| norm activation model (nam) | norm activation model |
| norm activation model theory | norm activation model |
| norm activation theory | norm activation model |
| norm-activation theory | norm activation model |
| norm-activation-theory | norm activation model |
| normative activation theory | norm activation model |
| organizations | organization |
| pandemics | pandemic |
| perceived behavioural control | perceived behavioral control |
| perceived corporate social responsibility (csr) | perceived corporate social responsibility |
| personal norm | personal norms |
| planned behavior theory | theory of planned behavior |
| planned behaviour | theory of planned behavior |
| pls | pls-sem |
| private cars | private car |
| pro-environmental behaviour | pro-environmental behavior |
| pro-environmental behavior (peb) | pro-environmental behavior |
| pro-environmental behaviors | pro-environmental behavior |
| pro-environmental intentions | pro-environmental intention |
| pro-environmental intent | pro-environmental intention |
| pro-social behaviour | pro-social behavior |
| pro-social intentions | pro-social intention |
| pro-social behavioral intentions | pro-social intention |
| purchase intentions | purchase intention |
| social norm | social norms |
| stimulus-organism-behaviour-consequences theory | stimulus-organism-behavior-consequences theory |
| structural equation modeling | structural equation model |
| structural equation modeling (sem) | structural equation model |
| structural equation modelling | structural equation model |
| structural equations | structural equation model |
| subjective norm | subjective norms |
| sustainable consumption behavior | sustainable consumption |
| sustainable transport | sustainable transport behavior |
| the norm activation model | norm activation model |
| the norm activation model (nat) | norm activation model |
| theory of planned | theory of planned behavior |
| theory of planned behavior (tpb) | theory of planned behavior |
| theory of planned behaviour | theory of planned behavior |
| the theory of planned behavior | theory of planned behavior |
| the theory of planned behavior (tpb) | theory of planned behavior |
| tpb | theory of planned behavior |
| transport policy | transportation policy |
| travel-mode choice | travel mode choice |
| urban transport | urban transportation |
| value-belief-norm theory | value belief norm theory |
| value-belief-norm-theory | value belief norm theory |
| voluntary pro-environmental behavior (vpeb) | voluntary pro-environmental behavior |
| waste classification | waste sorting behavior |
| waste separation behavior | waste sorting behavior |
| waste separation behaviour | waste sorting behavior |
| waste sorting | waste sorting behavior |
| waste sorting behaviour | waste sorting behavior |
| willingness-to-pay | willingness to pay |
| willingness to pay (wtp) | willingness to pay |
| willingness to consume | willingness to pay |
| zero-waste living | zero-waste lifestyle |
| england | united kingdom |
| scotland | united kingdom |
| wales | united kingdom |
| taiwan | china |
| peoples r china | china |
| u arab emirates | united arab emirates |
